# Editorial: Social interaction in cyberspace: online gaming, social media, and mental health

**DOI:** 10.3389/fpsyt.2026.1843214

**Published:** 2026-05-19

**Authors:** Cody Ding, Xuemei Gao, Antao Chen, Xiaojun Sun

**Affiliations:** 1 Department of Education Sciences and Professional Programs, University of Missouri-St. Louis, St. Louis, MO, United States; 2 Southwest Jiaotong University, Chengdu, China; 3 Shanghai Jiaotong University, Shanghai, China; 4 Central China Normal University, Wuhan, China

**Keywords:** social media, cyberspace, mental health, problematic digital use, online social interaction

This Research Topic brings together interdisciplinary scholarship examining how digitally mediated social interaction shapes psychological wellbeing, vulnerability, and adaptation across diverse populations and contexts. Collectively, the contributions show that cyberspace is not merely a backdrop for social behavior, but an active environment in which identity, belonging, stress, support, and risk are produced and negotiated. Recent guidance and reviews likewise emphasize that the effects of social media and related digital technologies are not inherently beneficial or harmful; rather, outcomes depend on developmental stage, individual characteristics, patterns of engagement, and the specific affordances and content of platforms (1–3).

Digital technologies now constitute a central infrastructure of everyday social life. For adolescents and young adults in particular, interaction increasingly unfolds through social media, short-video platforms, smartphones, and other networked environments that shape emotional expression, affiliation, self-presentation, and coping. These developments have raised pressing questions for psychiatry and mental health research. On the one hand, digital environments may facilitate connectedness, information exchange, social support, and resilience. On the other hand, they may contribute to compulsive use, social comparison, loneliness, interpersonal strain, and exposure to harmful content. Recent advisories from the American Psychological Association and the U.S. Surgeon General both argue for a balanced but precautionary approach, stressing potential benefits alongside clear concern about harms, especially for youth who may be vulnerable because of developmental stage or pre-existing mental health difficulties (1, 3). The papers assembled in this Research Topic demonstrate with considerable nuance that digital life is neither uniformly beneficial nor uniformly harmful. Rather, its consequences depend on the interaction among individual vulnerabilities, social contexts, platform affordances, and the meanings users assign to online engagement.

A major strength of this Research Topic is that it moves beyond reductive accounts of “screen time” and instead focuses on the mechanisms through which digital engagement becomes adaptive or maladaptive. Several papers address problematic or addictive forms of use. This emphasis is well aligned with recent reviews showing that how young people use social media, and the feedback, comparison, and emotional processes involved, often matter more than time alone (2, 4, 5). In When time slows down: Temporal distortion and addictive social media use, temporal distortion is proposed as a potentially important process in addictive social media use, drawing attention to how altered subjective time perception may undermine self-regulation and sustain excessive engagement. (Vinci et al.) A complementary conceptual advance appears in Placing problematic media use in context: a research synthesis, person-centric framework, and chart review among a clinical sample of US youth, which introduces the Person–Context–Process–Outcome–Time model and frames problematic media use as a cyclical, context-sensitive, and developmentally embedded phenomenon. (Carter et al.) Together, these contributions help shift the field from descriptive concern about digital overuse toward more clinically meaningful accounts of how dysregulated patterns emerge and persist.

The empirical studies in this Topic further show that problematic digital engagement is often linked to emotional distress rather than to digital behavior in isolation. In Perceived social support and social/non-social problematic smartphone use among Chinese university students: a cross-sectional study of indirect associations via social anxiety, perceived social support was indirectly related to problematic smartphone use through social anxiety. (Liu et al.) Similarly, Mobile phone addiction and achievement motivation in Chinese medical students: the chain mediating effect of anxiety and depression symptoms identifies anxiety and depression symptoms as mediators linking mobile phone addiction with reduced achievement motivation. (Bai et al.) This pattern is consistent with recent literature showing that the mental health correlates of social media and smartphone use are shaped by factors such as social comparison, fear of missing out, cyberbullying, sleep disruption, and pre-existing vulnerability, rather than by exposure alone (1, 5, 6).

The Research Topic also offers important insight into loneliness and social support in online settings. Digital platforms are often presented as remedies for social isolation, yet the relationship between online support and wellbeing is clearly more complex. In From lonely to addicted: exploring sex differences in the effect of online social support among university students in Hong Kong, online social support partially mediated the association between loneliness and internet addiction among male students, suggesting that support acquired online may, in some cases, reinforce dependence on digital environments rather than alleviate underlying disconnection. (Leung et al.) This tension resonates with Digital loneliness as a new diagnostic category in psychiatry: the impact of technology and social media use on psychological wellbeing, which associates greater social media use with higher loneliness and anxiety, particularly among younger users. (Szawarnoga et al.) More broadly, recent literature suggests that active, socially connecting forms of online engagement may at times be beneficial, whereas comparison-driven, appearance-focused, or feedback-dependent use is more likely to be harmful (1, 4).

At the same time, the Research Topic avoids a one-sided deficit model of online life. From perceived threat to coping strategies: exploring the role of social media and its impact on loneliness and anxiety during the COVID-19 quarantine offers an important counterpoint, showing that social media can help mitigate loneliness and anxiety during crises, particularly by enhancing social support and resilience. (Jin et al.) This interpretation is consistent with APA guidance, which notes that online interaction can promote companionship, emotional intimacy, and social support, especially during periods of stress or social isolation (1). This contribution serves as a reminder that digital platforms can function as tools of adaptation under conditions of uncertainty and social disruption. The more productive question for psychiatry, therefore, is not whether social media is inherently beneficial or harmful, but under what circumstances it supports coping, connectedness, and recovery rather than exacerbating vulnerability.

Several papers in the Topic also demonstrate that communicative practices and platform-specific norms are central to understanding mental health outcomes online. In Distress disclosure on social media and depressive symptoms among college students: the roles of social comparison and gender, the association between distress disclosure and depressive symptoms is mediated by social comparison, with gender shaping the strength of this pathway. (Ye et al.) Likewise, Digital stress and friendship conflict in adolescence: the role of perceived norms and features of social media shows that expectations of online availability, perceived norms, and platform features contribute to digital stress and friendship conflict in adolescence. (Angelini et al.) These findings fit with recent reviews and advisories highlighting the importance of specific platform features, such as endless scrolling, feedback metrics, algorithmic recommendations, and constant availability, in shaping young users’ expeiences (1–3).

This attention to platform context becomes especially important in Digital mental health and hidden support: a qualitative analysis of non-suicidal self-injury communities on TikTok. (Martinez-Pastor et al.) By documenting euphemisms, coded hashtags, aesthetic cues, and limited moderation, this study shows how sensitive mental health content may circulate through indirect and socially meaningful forms of communication that are difficult to regulate. Importantly, the paper avoids simplistic interpretations: the communities examined function not only as spaces in which harmful behaviors may be normalized, but also as sites of recognition and hidden support. Recent literature similarly notes concern about harmful exposure, contagion processes, and platform-mediated amplification, while also stressing that the evidence base remains methodologically challenging and often too coarse to support simple causal claims (2, 5).

Another notable strength of the Research Topic is its contribution to conceptual and methodological development. Research on digital technology use has long relied on narrow indicators, often emphasizing duration or frequency at the expense of lived experience. New measurements of digital technology use: the Immersion in Digital Life and Quality of Digital Experience scales address this limitation by introducing measures that assess wellbeing, time and efficiency, social connectedness, and the broader extent to which digital technology permeates everyday life. (Witowska et al.) Validation of the immersion in digital life and quality of digital experience scales in German, French, Spanish, Polish, and Czech extends this contribution across multiple linguistic and cultural settings. (Witowska et al.) These papers are especially timely because recent critiques argue that stronger measurement, clearer theory, and better causal methods are essential if the field is to move beyond inconsistent associations and policy controversy toward a more reliable evidence base (2, 3).

The Topic also points toward modifiable targets for prevention and intervention. The relationship between physical activity and short video addiction among college students: mediating effects of self-control and social anxiety suggests that physical activity may reduce short-video addiction both directly and indirectly through improved self-control and reduced social anxiety. (Wang et al.) This finding is particularly valuable because it moves the discussion from risk description to actionable protective processes. Across the Research Topic more broadly, resilience, emotional regulation, social support, and self-control repeatedly emerge as promising leverage points for prevention, clinical intervention, and digital health promotion. This emphasis is consistent with recent clinical literature suggesting that healthier digital engagement may be fostered not only by restriction, but also by building intrapersonal and interpersonal skills and by developing targeted interventions for problematic social media use (4, 7).

Finally, Stock and cryptocurrency trading and problem gambling behavior during early phases of the COVID-19 pandemic: A narrative literature review broadens the scope of the Research Topic beyond social networking and gaming alone. (Wei Lyn et al.) By highlighting excessive online trading as a behavior sharing important features with problem gambling, this review reminds us that digital psychiatry must attend to a wider range of reward-driven and potentially addictive online activities. This broader lens is especially timely in an era when financial speculation, entertainment, algorithmic recommendation, and social influence increasingly converge within digital ecosystems.

Taken together, the 14 articles in this Research Topic converge on several key conclusions. First, the mental health effects of cyberspace are mediated by psychological and relational processes, including anxiety, depression, loneliness, resilience, self-control, social comparison, and perceived social support. Second, platform affordances and social norms matter: visibility, responsiveness, coded communication, and expectations of constant availability all shape user experience. Third, benefits and harms frequently coexist within the same digital environments. Online interaction may provide support, belonging, and coping, even as it intensifies dependency, distress, or interpersonal conflict. Fourth, theoretical refinement and improved measurement are indispensable if the field is to move beyond descriptive association toward clinically useful understanding (1, 2, 5).

Overall, the studies collected here advance a more mature and balanced understanding of social interaction in cyberspace. By combining conceptual innovation, psychometric development, qualitative inquiry, and empirical research across diverse settings and populations, this Research Topic underscores the need for psychiatry to engage digital life not as a peripheral issue, but as a central domain of contemporary mental health. We hope that this Research Topic will stimulate further interdisciplinary research and inform interventions to foster digital environments that are not only technologically engaging but also psychologically safer and more supportive of human flourishing.
